# Editorial: ROS Regulation during Plant Abiotic Stress Responses

**DOI:** 10.3389/fpls.2016.01536

**Published:** 2016-10-19

**Authors:** Zhulong Chan, Ken Yokawa, Woe-Yeon Kim, Chun-Peng Song

**Affiliations:** ^1^Key Laboratory of Plant Germplasm Enhancement and Specialty Agriculture, Wuhan Botanical Garden/Sino-Africa Joint Research Center, Chinese Academy of SciencesWuhan, China; ^2^Institute of Cellular and Molecular Botany, University of BonnBonn, Germany; ^3^Department of Biological Sciences, Tokyo Metropolitan UniversityTokyo, Japan; ^4^Division of Applied Life Science (BK21 Plus), Plant Molecular Biology and Biotechnology Research Center, Institute of Agriculture and Life Sciences, Gyeongsang National UniversityJinju, South Korea; ^5^Department of Biology, Henan UniversityKaifeng, China

**Keywords:** abiotic stress, hormones, reactive oxygen species, redox

Plants frequently encounter a combination of abiotic stresses in their natural habitats. Abiotic stresses, including drought, salt, cold, heat, and heavy metal etc., modulate phytohormone metabolism and enhance expression level of transcription factors which activate stress responsive genes. During plant stress response, reactive oxygen species (ROS) act as important molecules and play pivotal roles in activating downstream metabolic pathways. In this Research Topic, we collected 25 manuscripts related to ROS and redox regulation in plant responses to abiotic stress, including reviews of the role of ROS in plant abiotic stress responses and articles related to gene function analysis, genome-wide gene expression and transcriptomic analysis, and interaction analysis between ROS and phytohormones.

Krieger-Liszkay and Feilke reviewed how plastid terminal oxidase (PTOX) interplays with the photosynthetic electron flow and hypothesized that the function of PTOX is dependent of stromal pH. Corpas and Barroso briefly summarized possible roles of reactive sulfur species (RSS) in peroxisomes and hypothesized potential interactions among ROS, reactive nitrogen species (RNS) and sulfur-containing compounds. In crop plants, ROS regulation under abiotic stress condition has been reviewed by You and Chan. In response to abiotic stress, plants have evolved complex signaling pathways to regulate sets of stress responsive genes encoding protein kinases, phosphatases, transcriptional factors, SIMILAR TO RCD ONE (SRO) proteins, ROS-scavenging or detoxification proteins, and proteins involved in hormone pathway and calcium signal (You and Chan). Sewelam et al. summarized that ROS functions as the primary source of the signaling battery in plants under stressed conditions and may interact with other signaling components, e.g., calcium, redox homeostasis, membranes, G-proteins, MAPKs, plant hormones, and transcription factors. Sewelam et al. discussed the interaction between nitric oxide (NO) and ROS which might regulate abscisic acid (ABA) biosynthesis to modulate stomatal closure. Liu et al. reviewed the functions of polyamines (PAs) during plant stress response through modulation of antioxidant systems or suppression of ROS production. Evidences showed that PA catabolism resulted in the production of H_2_O_2_. Exogenous supply of PAs or ectopic expression of PAs biosynthesis related genes increased antioxidant system in several plants (Liu et al.). Dinakar et al. demonstrated the importance of AOX pathway in optimizing photosynthesis in *Pisum sativum* in the presence of osmotic and temperature stress conditions. These articles summarized roles of ROS during plant stress response and possible interaction of ROS with hormones and other chemicals.

Compared to extensive studies on drought, cold, heat, and osmotic stresses, less attention has been paid to heavy metal stress and light stress, which become increasingly important stress factors limiting plant growth. Three research articles addressed aluminum (Al), cadmium (Cd) and UV-B stress responses, respectively. Lin et al. observed that Al^3+^ stress induced ${\text{O}}_{2}^{-•}$ generation in the cell suspension cultures of tobacco and rice, while pretreatment with various concentration of Zn^2+^ significantly inhibited the Al^3+^-induced oxidative burst. Moreover, Al^3+^-induced cell death was also inhibited in the presence of Zn^2+^. High concentration of zinc (0.5 mM) effectively lowered the level of Al^3+^-induced [Ca^2+^]_*c*_ elevation. In tomato, cadmium (Cd) stress significantly inhibited activities of superoxide dismutase (SOD), catalase (CAT) and peroxidase (POD), while increased the contents of H_2_O_2_ and ${\text{O}}_{2}^{-•}$, resulted in increased malondialdehyde (MDA) and electrolyte leakage (EL) (Hasan et al.). Yokawa et al. found that UV-B promoted the robust generation of ROS which affects endocytic vesicle recycling in Arabidopsis root apex. It is well known that many root tropisms require elaborate control of endocytic vesicle recycling in the cells. This finding explains that how light stress situation triggers root negative phototropism through ROS production as a rapid signaling event.

Omic approaches were effectively applied to identify genes involved in plant stress responses. de Abreu Neto and Frei conducted a meta-analysis of microarray experiments in rice. Publicly available microarray transcriptome data were re-analyzed. The results showed that ROS-related genes were overrepresented among the differentially expressed genes (DEGs). After treatments with oxidative stress (ozone and H_2_O_2_) and abiotic stresses, 990 and 1727 shared DEGs were identified, respectively. Among them, 311 genes were overlapped by both oxidative and abiotic stresses and 33 were ROS-related genes. Additionally, Mata-Pérez et al. identified genes in response to linolenic acid, a precursor of jasmonic acid (JA) using RNA-seq approach. In total, expression levels of 3034 genes were changed after linolenic acid treatment. This study showed that linolenic acid modulated the expression of genes involved in stress response, particularly those mediated by ROS signaling. Several transcription factors including *WRKY, JAZ, MYC* were also modified in response to linolenic acid. These data indicated that abiotic stress modulated expression of stress responsive genes as well as ROS related genes. Wei et al. identified 85 *WRKY* genes in cassava (*Manihot esculenta*) through bioinformatics analysis. RNA-seq data showed that 78 *MeWRKY* genes were differentially expressed in response to drought stress and 9 *MeWRKY* genes were modulated after NaCl, mannitol, cold, H_2_O_2_ and ABA treatments, indicating that *MeWRKY* genes were involved in plant stress response and redox signaling pathway. These analyses provided new clues for identification of genes involved in oxidative stress.

Detailed functions of several stress responsive genes including transcription factors have been characterized. The Universal Stress Protein domain (USP) gene modulated plant response to a wide variety of abiotic stresses. The biochemical function of *AtUSP* (At3g53990) was characterized by Jung et al. The results showed that *AtUSP*-OX plants were tolerant to heat shock and oxidative stresses, whereas the knock-out mutants were sensitive to the stress treatments. *AtUSP* exhibited a redox-dependent chaperone function which might contribute to its protective roles during diverse stress conditions. The Arabidopsis sulfotransferase gene *AtSOT12* is a salt inducible gene through transcriptome analysis. Chen et al. found that salt stress affected capping and polyadenylation of *AtSOT12*, but not DNA methylation level in the promoter region. Expression of *AtSOT12* was induced by salt stress is partially through ABA-INSENSITIVE 1 (ABI1)—and SALT OVERLY SENSITIVE 1 (SOS1)-mediated signaling pathways. Mutation of oxidative stress related *oxi1* resulted in increased *AtSOT12* expression, while ROS scavenger treatments also enhanced *AtSOT12* transcript level, indicating that ROS production might be involved in the repression of *AtSOT12* gene. Baek et al. isolated an Arabidopsis *ars1* (*aba and ros sensitive 1*) mutant which showed hypersensitivity to ABA and methyl viologen (MV). *ARS1* encodes a nuclear protein with one zinc finger domain. Expression level of *CSD3* gene encoding SOD was reduced and ROS was accumulated in *ars1* mutant. Furthermore, *ARS1* inhibited ABA-induced ROS production. Treatment with ABA, H_2_O_2_ and MV modulated localization of ARS1 protein. Wang et al. assembled 10 *WRKY* unigenes from ESTs of wheat (*Triticum aestivum*). Among them, *TaWRKY44* was upregulated by various stress treatments, hormones, and H_2_O_2_. TaWRKY44 localizes to the nucleus and binds to the core DNA sequences of TTGACC and TTAACC in yeast. *TaWRKY44* transgenic tobacco plants showed increased drought and salt tolerance. Under osmotic stress condition, transgenic lines exhibited lower H_2_O_2_ content and higher SOD, CAT, and POD activities. Overexpression of *TaWRKY44* increased expression of several ROS related genes and stress-responsive genes. The results indicated that several transcription factors might function as ROS upstream regulators.

In response to environmental stresses, plants develop various strategies, including induction of phytohormones. Among the, auxin regulates plant growth and development. YUCCA6, a flavin monooxygenase enzyme, converts indole-3-pyruvic acid to auxin. Cha et al. reported that overexpression of *YUCCA6* in Arabidopsis reduced the expression of senescence related gene *SAG12* and delayed leaf senescence. *YUCCA6*-OX plants, but not mutated *YUCCA6*-OX^C85S^, a dysfunctional mutation of ROS homeostasis maintained by YUCCA6, exhibited reduced ROS accumulation and increased expression of genes encoding NADPH-dependent thioredoxin reductases and *GSH1* involved in redox signaling. Moreover, auxin efflux proteins at both transcriptional and protein level were reduced either by ROS balance or by thiol-reductase activity of YUCCA6. Additionally, overexpression of the cytokinin biosynthetic gene *AtIPT8* (adenosine phosphate-isopentenyltransferase 8) in *Arabidopsis thaliana* resulted in increased endogenous cytokinin content (Wang et al.). *AtIPT8* transgenic lines showed increase sensitivity to salt stress and accumulated higher ROS content than the wild type control. Moreover, many genes involving in photosynthesis and abiotic stress responses were differentially expressed in *AtIPT8* transgenic lines (Wang et al.). Therefore, phytohormones affected plant stress responses partially through modulation of ROS levels.

Exogenous application of hormones and chemicals increased plant tolerance to various abiotic stresses. Treatment with spermidine promoted the growth recovery of rice after drainage. Spermidine treatment decreased ROS generation and improved photosynthesis in submerged rice. Addition of polyamine (PA) alleviated the suppressing effects of osmotic stress in leaves of white clover. Further study showed that PA was involved in regulation of H_2_O_2_ and Ca^2+^ messenger. PA-induced H_2_O_2_ production required Ca^2+^ release, while PA-induced Ca^2+^ release was also essential for H_2_O_2_ production (Li et al.). Pretreatment with spermidine in rice improved submergence tolerance through modulation of ROS production and chlorophyll degradation (Liu et al.). Xu et al. found that exogenous ascorbic acid treatment improved root growth in tall fescue (*Festuca arundinacea*) under water stress condition. Roots in ascorbic acid (ASA)-treated plants had lower ROS and MDA contents, higher non-enzymatic antioxidant accumulation, and increased expression of genes encoding cell-wall loosening proteins. Melatonin (N-acetyl-5-methoxytryptamine) has long been known to be an important animal hormone and identified in various plant species since 1995. In tomato (*Solanum lycopersicum*), cadmium (Cd) stress significantly increased the contents of Cd and melatonin (Hasan et al.). Exogenous application of melatonin increased activities of antioxidant enzymes and H^+^-ATPase, and contents of glutathione (GSH) and phytochelatins. Supplementation with melatonin significantly reduced leaf Cd accumulation. In common wheat (*Triticum aestivum*), application of ABA caused decreased contents of H_2_O_2_ and MDA and increased GSH and ASA under osmotic stress condition (Wei et al.). Gene expression analysis showed that ABA treatment regulated transcripts of genes encoding ASA and GSH synthesis-related enzymes. The results shed lights on exogenous application of chemicals to improve plant stress tolerance.

Under abiotic stress condition, metabolites like dehydrin and proline appear to function in stress tolerance by serving as a compatible solute or osmoprotectant. Dehydrin belongs to group II late embryogenesis abundant protein (LEA). Shi et al. characterized functions of Arabidopsis *LOW TEMPERATURE-INDUCED 30* (*LTI30*), encoding a LEA protein, under drought stress condition. *AtLTI30* knockout mutant was less sensitive to ABA and displayed decreased drought tolerance, whereas *AtLTI30*-OX plants were more sensitive to ABA and showed improved drought tolerance. Manipulation of *AtLTI30* expression increased activities of CAT and decreased drought stress-triggered H_2_O_2_ production. In trifoliate orange (*Poncirus trifoliate*), Peng et al. cloned a hybrid proline-rich protein gene *PtrPRP*. Expression level of *PtrPRP* was progressively induced upon cold stress treatment. *PtrPRP* knock-down lines displayed sensitivity to cold stress as evidenced by higher EL, MDA content, and increased accumulation of ROS. In aged oat seed, Kong et al. suggested that proline and antioxidant enzymes played the main role in adaptation to oxidative stress in seeds with higher (28%) and lower (4%, 16%) moisture contents, respectively. These studies highlighted the protective roles of osmoprotectants and putative functions in modulating of ROS and redox pathways.

In summary, we focus on the roles of ROS during plant abiotic stress responses in this Research Topic. Plant responses to multiple abiotic stresses and effects of hormones and chemicals on plant stress responses have been carefully studies. Although functions of several stress responsive genes have been characterized and possible interactions between hormones and ROS are discussed, future researches are needed to functionally characterize ROS regulatory and signaling transduction pathways.

## Author contributions

ZC and CS wrote the manuscript, KY and WK added notes and revised the manuscript.

### Conflict of interest statement

The authors declare that the research was conducted in the absence of any commercial or financial relationships that could be construed as a potential conflict of interest.

